# Social Determinants of Digital Health Intervention Use in China: National Cross-Sectional Study

**DOI:** 10.2196/94788

**Published:** 2026-08-21

**Authors:** Huohuo Dai, Yibo Wu, Anke Versluis, Yiling Li, Niels Chavannes, Jiska Aardoom

**Affiliations:** 1 Department of Public Health and Primary Care, Leiden University Medical Center, Hippocratespad 21, Leiden, South Holland, 2333 ZD, The Netherlands; 2 National eHealth Living Lab, Leiden University Medical Center, Leiden, South Holland, The Netherlands; 3 Department of nursing, The Fourth Affiliated Hospital of School of Medicine, International school of medicine, and International Institutes of Medicine, Zhejiang University, Yiwu, Zhejiang, China; 4 School of Health Policy and Management, Chinese Academy of Medical Sciences & Peking Union Medical College, Beijing, Beijing, China

**Keywords:** digital health interventions, health equity, social determinants of health, digital divide, health care access, eHealth literacy, China

## Abstract

**Background:**

Digital health interventions (DHIs), including telemedicine and AI-enabled health tools, are increasingly integrated into health care systems worldwide. While these technologies have the potential to improve access and efficiency, unequal access to resources and health capabilities may create disparities in their use. Evidence remains limited on how social and structural determinants shape population-level entry into DHI use in rapidly digitalizing health systems such as China.

**Objective:**

This study aimed to examine social and structural determinants of broader DHI use among adults in China using the World Health Organization’s (WHO) Social Determinants of Health (SDoH) framework.

**Methods:**

This cross-sectional study analyzed data from a national survey conducted in China in 2024 among adults aged 18 years or older. The primary outcome was self-reported ever use of DHIs, including telemedicine, digital health apps, and AI-enabled health tools. Explanatory variables were categorized into 5 SDoH domains: economic stability, education and health-related capabilities, health care access and quality, neighborhood and built environment, and social and community context. Multivariable logistic regression was used to estimate adjusted odds ratios (aORs) and 95% CIs. Adjusted predicted probabilities and subgroup analyses by age and region were conducted as supplementary analyses.

**Results:**

Among 34,672 participants, 14,565 (42.0%) reported ever using a DHI. DHI use was higher among participants with greater socioeconomic resources and health-related capabilities, including higher income, higher educational attainment, and higher health and eHealth literacy (HL, eHL). For example, participants with a monthly income of 6001 CNY (approximately US $889) or higher had higher odds of DHI use than those with an income of 3000 CNY (approximately US $444) or less (aOR 1.370, 95% CI 1.285‐1.462), and those with a bachelor’s degree or above had higher odds than those with junior high school education or below (aOR 1.487, 95% CI 1.377‐1.607). Health system engagement also showed substantial differences: participants unaware of family doctor services had lower odds of DHI use than those enrolled in such services (aOR 0.502, 95% CI 0.465‐0.542). Difficulty paying medical expenses was associated with higher DHI use (aOR 1.314, 95% CI 1.221‐1.414). Adjusted predicted probabilities showed meaningful absolute differences for education, income, HL, eHL, difficulty paying medical expenses, and family doctor service status, whereas the adjusted urban-rural difference was small.

**Conclusions:**

Broader self-reported DHI use in China is strongly associated with socioeconomic resources, health-related capabilities, and access to health care. Equitable digital health transformation requires more than technological expansion. Strategies to improve digital health equity should strengthen HL and eHL, integrate digital tools with accessible primary care and family doctor services, preserve nondigital support pathways, and incorporate equity-oriented design and governance as AI-enabled health tools become more widely implemented.

## Introduction

Digital technologies have been rapidly adopted in health care and play a significant role in extending universal health coverage, improving emergency resilience, and enhancing well-being [1]. The COVID-19 pandemic, coupled with the emergence of innovations such as large language models, has further accelerated the global adoption of digital health technologies [1]. In parallel, global policies such as the World Health Organization’s (WHO) Global Digital Health Strategy [1] and the Global Initiative on AI for Health (GI-AI4H) [2] have reinforced the need to implement digital health solutions in ways that are effective, safe, and equitable.

Digital health interventions (DHIs) refer to distinct technological functions or capabilities specifically developed to address particular challenges within health care systems [3]. They offer substantial potential to improve health outcomes due to their scalability, cost-effectiveness, and potential for efficient resource allocation [1,4-7]. However, the equity implications of this rapid expansion remain uncertain. The benefits of DHIs depend not only on whether such technologies are available or technically effective, but also on whether different population groups are able to access, understand, trust, and meaningfully use them.

Digital health inequities are therefore more than a problem of unequal access to devices or internet connectivity [8-10]. As digital health services become increasingly embedded in routine health care, disparities may arise from differences in financial resources, digital access, health literacy, eHealth literacy (eHL), social support, and health care access. These barriers may be particularly salient among older adults and populations in underserved regions and may contribute to widening health inequities in the digital era [8,9]. Inequity-related barriers operate at multiple levels, including individual-level factors such as sex, age, digital literacy, and internet access [11,12]; community characteristics such as living region and community resources [11,13]; and broader societal conditions such as health policy and health insurance [9,14]. In this sense, DHI use reflects both first-level digital divides related to access and second-level divides related to skills, capabilities, and meaningful engagement. As a result, populations already facing socioeconomic and structural disadvantages may be less likely to benefit from digital health transformation and may be disproportionately affected if health systems increasingly rely on digital pathways [8,9,15]. Understanding the social patterning of DHI use is essential for designing digital health strategies that reduce rather than reproduce health inequities.

China provides an important setting for examining these issues. National policies have strongly supported the expansion of digital health, and digital platforms, internet-based health care services, wearable devices, and AI-supported health tools have become increasingly visible within the health system [16]. At the same time, China’s vast geography, large, diverse population, and uneven distribution of socioeconomic resources, health care infrastructure, and digital capacity create substantial challenges for ensuring equitable use of DHIs [13,17]. These conditions make China a critical context in which to examine whether digital health engagement is socially and structurally patterned during a period of rapid technological transformation.

Previous studies have examined internet access and mobile phone ownership, internet use and health inequalities, and internet medical services used in China [13,18,19]. However, these studies have mainly focused on access to digital technologies, general internet use, specific internet-based medical services, or selected population groups such as older adults or patients. Less is known about broader self-reported DHI use as a population-level indicator of entry into digital health pathways among adults in China. In particular, limited evidence has examined how social and structural conditions shape population-level entry into digital health use across socioeconomic, capability-related, health care access, geographic, and social-contextual domains in a postpandemic and increasingly AI-enabled digital health context.

The WHO Social Determinants of Health (SDoH) framework provides a useful structure for examining these multidomain influences. The framework emphasizes that health-related opportunities and outcomes are shaped by the conditions in which people are born, grow, live, work, and age [20]. Applied to digital health, this perspective shifts attention from whether individuals choose to use DHIs to how economic stability, education and health-related capabilities, health care access and quality, neighborhood and built environment, and social and community context may jointly shape opportunities for DHI use [21]. Using this framework allows for a comprehensive understanding of how multiple layers of social disadvantage and health care access are jointly associated with DHI use in a rapidly digitalizing health system.

Therefore, this study uses the SDoH framework to examine social and structural determinants of broader DHI use among adults in China. The research question is: How is DHI use distributed across population subgroups, and which socioeconomic, capability-related, health care access, geographic, and social-contextual factors are associated with DHI use? Using data from a recent large-scale national survey across all regions in China, this study aims to (1) describe the distribution of DHI use across population subgroups and (2) examine which selected multilevel factors are associated with DHI use. By focusing on population-level entry into digital health pathways, this study provides evidence to inform equity-oriented digital health policy, design, and implementation.

## Methods

### Study Design

This study used data from the 2024 Psychology and Behavior Investigation of Chinese Residents (PBICR) [22]. PBICR is an annually administered national survey using stratified and quota-based sampling, designed to capture the psychological and behavioral characteristics of the Chinese population. The cross-sectional design enabled rapid data capture to inform ongoing changes in China’s digital health landscape. The present analysis focused on factors associated with self-reported use of DHIs among adults in China. The study is reported in accordance with the STROBE (Strengthening the Reporting of Observational Studies in Epidemiology) guideline [23].

Data collection took place between June 23, 2024, and September 29, 2024. The survey covered 150 cities across 4 municipalities, 22 provinces, 5 autonomous regions, and 2 special administrative regions in China. Further details of the PBICR 2024 protocol and sampling procedures have been reported elsewhere [22].

### Sampling and Recruitment

PBICR 2024 used a stratified and quota-based sampling approach. Briefly, cities were first selected across provincial-level administrative regions to ensure broad geographic coverage [22]. Communities or villages were then selected within sampled cities, with consideration of the urban-rural distribution. Within each selected community, quota sampling was used to approximate the age and sex structure of the Chinese population [22].

Recruitment was conducted by trained investigators through local community health centers, neighborhood committees, and community-based survey sites. Investigators approached potential participants face-to-face, assessed eligibility, and invited eligible individuals to complete an electronic questionnaire via Wenjuanxing, a secure online survey platform. Participants could complete the questionnaire independently or with assistance from trained investigators when needed. For participants with limited mobility or difficulty using the online platform, investigators conducted one-on-one assistance and recorded responses on their behalf. This offline-to-online approach was intended to combine community-based recruitment with the efficiency and standardization of electronic data collection.

### Participants and Analytic Sample

Eligible participants were adults (aged ≥18 y) with permanent residency in China who could complete the questionnaire either independently or with assistance. Individuals were excluded if they had significant cognitive or psychiatric impairments, had previously taken part in PBICR or comparable research projects, or declined participation. The exclusion of individuals who had previously participated in PBICR or comparable projects was intended to reduce repeated participation, potential survey exposure effects, and duplication across the PBICR database.

### Measurements

#### Demographic and Health-Related Covariates

Demographic covariates included age, sex, ethnicity, religious belief, and marital status. Health-related covariates included self-rated health and the presence of chronic conditions. Self-rated health was assessed using a visual analogue scale ranging from 0 to 100, with higher scores indicating better perceived health. Chronic conditions were measured as a binary variable (yes or no) based on self-reported physician diagnoses.

#### Outcome of Self-Reported DHI Use

In this study, participants’ DHI use was assessed using the following questions: Have you ever used DHIs (DHIs refer to the use of digital technologies via internet platforms to support and improve health and health care services)? These interventions can take various forms, such as smart health monitoring devices (eg, smart bands and smartwatches), health management apps, telemedicine, and AI-powered health consultation tools (single choice: yes or no).

This item was designed as a broad screening indicator of any prior engagement with DHI. The survey did not collect separate information on specific DHI subtypes, frequency, duration, intensity, purpose, or quality of use. Therefore, the outcome should be interpreted as any self-reported DHI use rather than subtype-specific DHI adoption or sustained digital health engagement.

#### SDoH Factors

Guided by the SDoH Framework [20], explanatory variables were organized into five domains: economic stability, education, health care access, neighborhood and built environment, and social and community context.

Economic stability was assessed using monthly income (in Chinese Yuan; at the time of calculation, CNY 1 was equivalent to approximately US $0.148), employment status, debt status (yes or no), and housing ownership. Housing ownership was categorized as none, 1 property, 2 properties, or 3 or more properties, with an additional category for respondents who were unsure.

Education and health-related capabilities included educational attainment, health literacy, and eHL. Health literacy was assessed using the short form of the Health Literacy Scale [24], developed from the European Health Literacy Questionnaire [25] and validated in Chinese populations. A standardized health literacy index was calculated using the established formula: (mean score−1) × (50/3), with higher scores indicating higher health literacy [24]. eHL represents the ability to search for, evaluate, and effectively use online health resources [26] and was measured using the Simplified eHealth Literacy Scale [26]. Responses were rated on a 5-point Likert scale, and the total score was the sum of the 3 dimensions, with a higher score indicating a higher eHL level.

Health care access was assessed using indicators of health insurance coverage (yes or no), difficulty paying medical expenses (yes or no), enrollment in family doctor services (yes, no, or not aware of the service), and the location of basic medical insurance enrollment (local residence, household registration location, or other cities).

Neighborhood and environmental characteristics included geographic region (eastern, central, or western China) and place of residence (urban or rural), which serve as broad indicators of contextual variation.

The social and community context was captured through perceived neighborhood relationship quality and social support. Neighborhood relationship quality was self-rated on a 7-point scale, with higher scores indicating better perceived relationships. Perceived social support was measured using the short version of the Perceived Social Support Scale, derived from the multidimensional Perceived Social Support Scale [27,28]. Each item is scored on a 7-point scale, and total scores are summed, with higher scores indicating higher perceived social support.

### Data Quality Control

The investigation implemented standardized quality control procedures throughout questionnaire development, investigator training, data collection, and questionnaire retrieval. Before formal data collection, the questionnaire was developed through literature review, expert consultation, and 3 rounds of presurvey testing; presurvey data were not included in the final analysis. Investigators and provincial coordinators received standardized training before fieldwork. During data collection, investigators verified participant eligibility face-to-face before questionnaire completion. Returned questionnaires underwent predefined quality checks, including completion-time screening, internal consistency checks, completeness checks, duplicate response detection, and assessment of abnormal response patterns [22]. Questionnaires failing these checks were excluded before construction of the analytic dataset. Further details of the quality control procedures are provided in the study protocol [22] and Multimedia Appendix 1.

### Missing Data

After eligibility screening, data quality checks, and study-specific exclusions, the final analytic dataset included participants with complete information on the study outcome and all covariates used in the regression models. Variable-specific missingness was therefore 0% for all model variables. No participants were excluded through complete-case analysis, and no imputation was performed.

### Statistical Analysis

Analyses were conducted using R version 4.5.1 (R Foundation for Statistical Computing). Descriptive statistics were first conducted to summarize the participants’ characteristics and the prevalence of DHI use across participant characteristics and regions. Categorical variables were summarized using frequencies and percentages, and continuous variables were summarized using medians and IQR ranges because their distributions were nonnormal.

Multivariable logistic regression was used to estimate associations between SDoH factors and DHI use. Variables were selected primarily based on the SDoH framework, prior literature, and availability in the survey. All theoretically relevant variables were entered simultaneously in the main model. Categorical variables were dummy-coded before analysis. All continuous variables were standardized (mean 0, SD 1), and effect estimates were interpreted per SD increase. Results were reported as adjusted odds ratios (aORs) with 95% CIs. Interpretation focused primarily on effect sizes, 95% CIs, direction, and consistency of associations, and absolute differences from adjusted predicted probabilities.

The primary regression analyses were conducted using the complete analytic sample without survey weights. Full design-based survey weighting was not applied because individual-level selection probabilities and design weights were not available for all sampling stages. Instead, the multivariable models adjusted for key demographic, socioeconomic, and health-related covariates to reduce potential confounding. However, this approach does not account for unequal sampling probabilities and should not be interpreted as a substitute for survey-weighted analyses. Several supplementary analyses were conducted to assess the robustness and interpretability of the findings. First, multicollinearity among model covariates was assessed using generalized variance inflation factors (GVIF), with adjusted GVIFs reported for categorical variables with more than 2 levels. Second, adjusted predicted probabilities and absolute differences were estimated for selected key determinants using marginal standardization to aid interpretation beyond odds ratios. Third, age- and region-stratified analyses were conducted to examine whether key associations were broadly consistent across population subgroups. These analyses involved multiple comparisons, and no formal adjustment for multiplicity was applied; therefore, the findings were interpreted descriptively and as hypothesis-generating rather than confirmatory. Details are provided in Multimedia Appendix 1.

### Ethical Considerations

Ethical approval was granted by the Ethics Review Committee of Shanghai Jiao Tong University (approval number: H202402371). All participants provided informed consent before participation. The questionnaire cover page explained the study purpose, the voluntary nature of participation, confidentiality and anonymity protections, and participants’ right to decline or withdraw. This study was conducted in accordance with the Declaration of Helsinki.

This study did not involve patients. The public was not involved in the design, reporting, or dissemination plans of our research. The privacy and confidentiality of the participants were protected during and after data collection.

## Results

### Participant Characteristics and Prevalence of DHI Use

**Figure 1.**
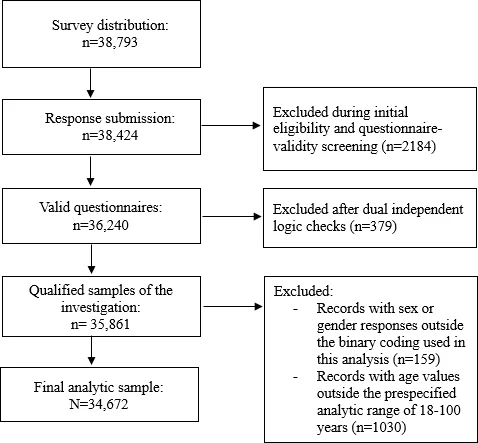
Flow diagram for inclusion of study participants. The 2184 questionnaires excluded during initial eligibility and questionnaire-validity screening included cases involving absence of informed consent, failure to meet residency or nationality eligibility criteria, and completion time of less than 5 minutes. Criterion-specific counts were not retained in the original centralized data-cleaning records; therefore, the verified aggregate count is reported.

**Figure 2.**
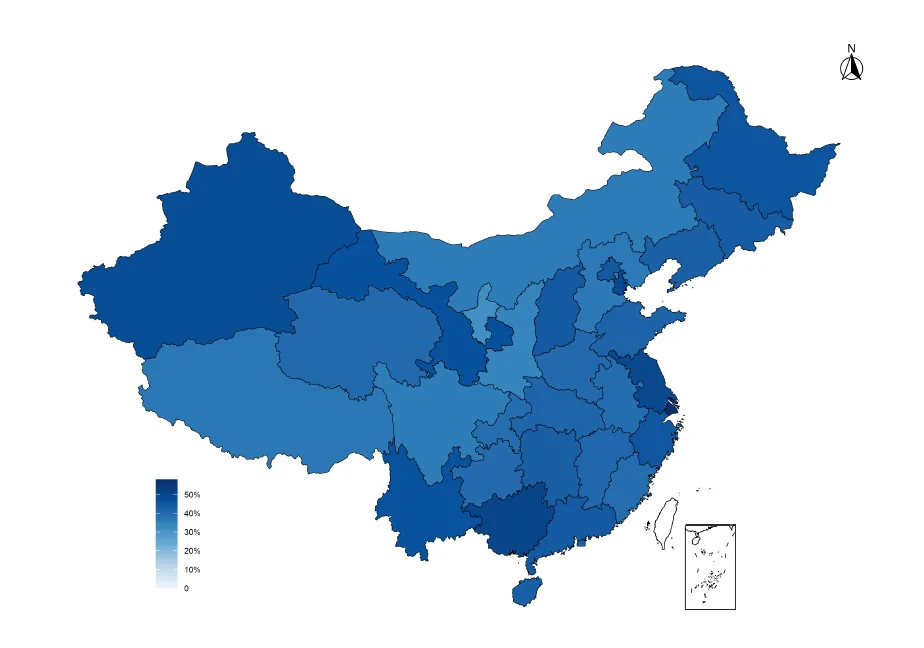
Digital health intervention (DHI) use rate across provinces in China. To improve the stability and comparability of provincial-level prevalence estimates, empirical Bayes (EB) smoothing was applied before mapping to reduce random variation arising from unequal sample sizes across provinces [29].

After applying eligibility criteria, data quality checks, and study-specific exclusions, 34,672 participants were included in the final analytic sample (Figure 1). Overall, 14,565 (42.0%) reported having ever used a DHI. Participant characteristics by DHI use status are summarized in Table 1. Compared with nonusers, DHI users were younger and more likely to reside in urban areas. DHI users also had higher socioeconomic indicators, including a greater proportion with a monthly income of 6001 or more and university education or above. Differences were also observed in health system engagement: participants reporting family doctor services were more common among DHI users, while the lack of awareness about family doctor services was less common. Regional distribution differed as well, with DHI users more frequently being from eastern China. Provincial variation was observed in empirical Bayes-smoothed DHI use rate (Figure 2).

**Table 1.** Sociodemographic and health characteristics of study participants, by digital health intervention (DHI) use status^a^.

Characteristics	No of participants (N=34,672), n (%)	DHI^b^ use status, n (%)	
		Yes (n=14,565)	No (n=20,107)
Age (y)			
18‐29	17,781 (51.28)	8415 (57.78)	9366 (46.58)
30‐44	6879 (19.84)	2873 (19.73)	4006 (19.92)
45‐59	6518 (18.80)	2224 (15.27)	4294 (21.36)
≥60	3494 (10.08)	1053 (7.23)	2441 (12.14)
Sex			
Female	19,657 (56.69)	8087 (55.52)	11,570 (57.54)
Male	15,015 (43.31)	6478 (44.48)	8537 (42.46)
Ethnicity^c^			
Han	31,130 (89.78)	13,171 (90.43)	17,959 (89.32)
Others	3542 (10.22)	1394 (9.57)	2148 (10.68)
Religious belief			
No	31,591 (91.11)	13,340 (91.59)	18,251 (90.77)
Yes	3081 (8.89)	1225 (8.41)	1856 (9.23)
Occupational status			
Employed (including freelance work)	15,934 (45.96)	6398 (43.93)	9536 (47.43)
Student	14,055 (40.54)	6712 (46.08)	7343 (36.52)
Retired	2879 (8.30)	928 (6.37)	1951 (9.70)
Unemployed	1804 (5.20)	527 (3.62)	1277 (6.35)
Residential type			
Urban	26,113 (75.31)	11,490 (78.89)	14,623 (72.73)
Rural	8559 (24.69)	3075 (21.11)	5484 (27.27)
Location of basic medical insurance enrollment^d^			
Local (current residence)	20,581 (59.36)	8841 (60.70)	11,740 (58.39)
Place of household registration	11,091 (31.99)	4357 (29.91)	6734 (33.49)
Other locations	3000 (8.65)	1367 (9.39)	1633 (8.12)
Difficulty paying medical expenses			
No	30,700 (88.54)	12,805 (87.92)	17,895 (89.00)
Yes	3972 (11.46)	1760 (12.08)	2212 (11.00)
Family doctor service^e^			
No, but aware of the service	16,730 (48.25)	7489 (51.42)	9241 (45.96)
Don’t know	13,997 (40.37)	4962 (34.07)	9035 (44.93)
Yes	3945 (11.38)	2114 (14.51)	1831 (9.11)
Number of properties			
1	17,669 (50.96)	6810 (46.76)	10,859 (54.01)
2	7409 (21.37)	3511 (24.11)	3898 (19.39)
None	3913 (11.29)	1543 (10.59)	2370 (11.79)
Not sure	3203 (9.24)	1338 (9.19)	1865 (9.28)
≥3	2478 (7.15)	1363 (9.36)	1115 (5.55)
Marital status			
Unmarried (single or divorced or widowed)	20,011 (57.72)	9406 (64.58)	10,605 (52.74)
Married	14,661 (42.28)	5159 (35.42)	9502 (47.26)
Chronic condition^f^			
No	27,911 (80.50)	12,036 (82.64)	15,875 (78.95)
Yes	6761 (19.50)	2529 (17.36)	4232 (21.05)
Monthly income (CNY; exchange rate: CNY 1 ≈ US $0.148.)			
3,001‐6000	14,361 (41.42)	5951 (40.86)	8410 (41.83)
≥6001	10,984 (31.68)	5388 (36.99)	5596 (27.83)
≤3000	9327 (26.90)	3226 (22.15)	6101 (30.34)
Debt status			
Yes	20,927 (60.36)	8632 (59.27)	12,295 (61.15)
No	13,745 (39.64)	5933 (40.73)	7812 (38.85)
Education level			
Bachelor’s degree or above	20,793 (59.97)	9833 (67.51)	10,960 (54.51)
High school or vocational school	7133 (20.57)	2962 (20.34)	4171 (20.74)
Junior high school or below	6746 (19.46)	1770 (12.15)	4976 (24.75)
Health insurance			
Yes	32,515 (93.78)	13,715 (94.16)	18,800 (93.50)
No	2157 (6.22)	850 (5.84)	1307 (6.50)
Region			
Central	13,571 (39.14)	5611 (38.52)	7960 (39.59)
Eastern	13,146 (37.92)	5877 (40.35)	7269 (36.15)
Western	7955 (22.94)	3077 (21.13)	4878 (24.26)

a Data are presented as n (%) unless otherwise indicated. Percentages are column percentages and may not sum to 100% due to rounding; no missing values were included in the denominators for variables shown in this table.

b DHI: digital health intervention.

c “Others” ethnicity refers to any non-Han ethnic group.

d Location of basic medical insurance enrollment: local (current residence); place of household registration; other locations.

e Family doctor service categories: yes, no, but aware of the service, or don’t know about the service.

f Chronic condition indicates self-reported chronic condition (yes or no).

### Social Determinants Associated With DHI Use

**Figure 3.**
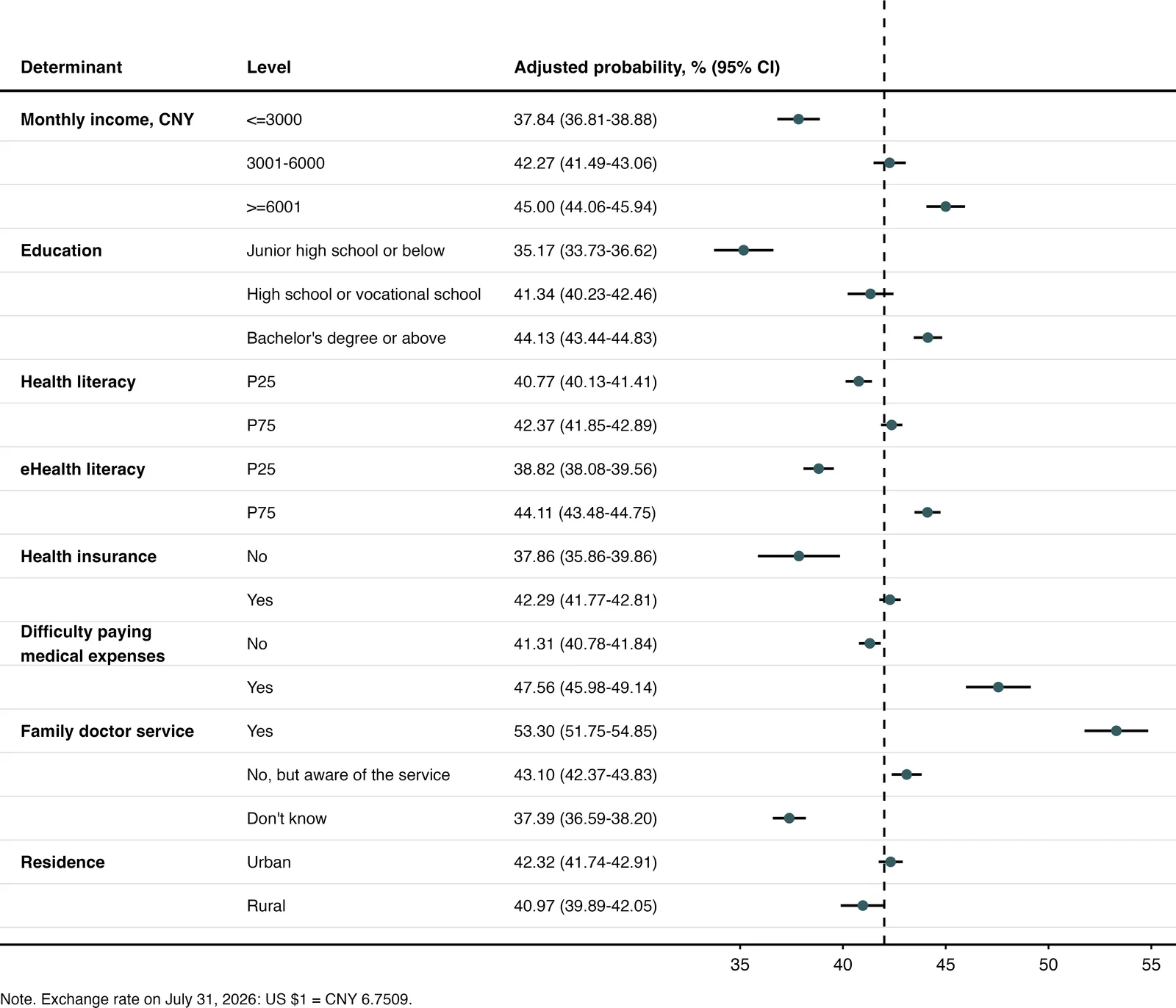
Adjusted predicted probabilities of digital health interventions (DHI) use for selected social determinants. The figure presents adjusted predicted probabilities and 95% CIs for any self-reported DHI use across selected social determinants. Estimates were derived from the primary unweighted multivariable logistic regression model using marginal standardization. The dashed vertical line indicates the overall prevalence of any self-reported DHI use in the analytic sample. P25 and P75 indicate the 25th and 75th percentiles for health literacy and eHealth literacy.

Adjusted associations between SDoH factors and DHI use are presented in Table 2 and adjusted predicted probabilities for selected determinants are shown in Figure 3. In the multivariable logistic regression model, older age was associated with lower odds of DHI use (aOR 0.926, 95% CI 0.887‐0.968), whereas male participants had higher odds (aOR 1.089, 95% CI 1.041‐1.139). Unmarried participants (single, divorced, or widowed) had higher odds of DHI use compared with married participants (aOR 1.327, 95% CI 1.238‐1.422). Presence of chronic conditions was positively associated with DHI use after adjustment (aOR 1.137, 95% CI 1.066‐1.214).

**Table 2.** Adjusted associations between social determinants of health and digital health intervention (DHI)^a^ use^b^.

Domain, characteristics, and classification	aOR^c^ (95% CI)
Sociodemographic factors (control variables)	
Age (per 1-SD increase)^d^	0.926 (0.887‐0.968)
Sex (reference: female)^e^	
Male	1.089 (1.041‐1.139)
Ethnicity (reference: others)	
Han	1.099 (1.012‐1.194)
Religious belief (reference: no)	
Yes	0.991 (0.913‐1.076)
Marital status (reference: married)	
Unmarried (single, divorced, or widowed)	1.327 (1.238‐1.422)
Health status (per 1-SD increase)	0.971 (0.947‐0.997)
Chronic condition (reference: no)	
Yes	1.137 (1.066‐1.214)
Economic stability	
Monthly income (reference: ≤3,000)	
3001‐6000	1.217 (1.149‐1.290)
≥6001	1.370 (1.285‐1.462)
Debt status (reference: no)	
Yes	0.975 (0.930‐1.022)
Occupational status (reference: employed [including freelance work])	
Student	1.002 (0.936‐1.074)
Unemployed	0.952 (0.848‐1.067)
Retired	1.119 (1.007‐1.245)
Number of properties (reference: none)	
1	1.068 (0.991‐1.152)
2	1.292 (1.187‐1.406)
≥3	1.492 (1.339‐1.664)
Not sure	1.054 (0.955‐1.164)
Education access and quality	
Education level (reference: junior high school or below)	
High school or vocational school	1.318 (1.214‐1.432)
Bachelor’s degree or above	1.487 (1.377‐1.607)
Health literacy index (per 1-SD increase)	1.100 (1.067-1.133)
eHealth literacy (per 1-SD increase)	1.202 (1.165‐1.240)
Health care access and quality	
Health insurance (reference: no)	1.219 (1.110‐1.340)
Yes	1.219 (1.110‐1.340)
Difficulty paying medical expenses (reference: no)	
Yes	1.314 (1.221‐1.414)
Family doctor service (reference: yes)	
No, but aware of the service	0.646 (0.600‐0.696)
Don’t know	0.502 (0.465‐0.542)
Location of basic medical insurance enrollment (reference: local [current residence])	
Place of household registration	0.938 (0.893‐0.986)
Other locations	0.987 (0.909‐1.070)
Neighborhood and built environment	
Region (reference: Eastern)	
Central	1.027 (0.975‐1.081)
Western	0.918 (0.862‐0.978)
Residential type (reference: urban)	
Rural	0.942 (0.891‐0.996)
Social and community context	
Social support (per 1-SD increase)	1.068 (1.042‐1.095)
Neighborhood relationship (per 1-SD increase)	0.978 (0.954‐1.002)

a DHI: digital health intervention.

b Estimates were obtained from the primary unweighted multivariable logistic regression model with DHI use (0=“no” and 1=“yes”) as the dependent variable.

c aOR: adjusted odds ratio.

d Continuous variables were standardized (mean 0, SD 1), and aORs for continuous variables are interpreted per 1-SD increase.

e For categorical variables, aORs are reported relative to the reference category indicated as “Reference.”

In the economic stability domain, compared with monthly income ≤3000, both income 3001‐6000 (aOR 1.217, 95% CI 1.149‐1.290) and income ≥6001 (aOR 1.370, 95% CI 1.285‐1.462) were associated with higher odds of DHI use. Compared with having no property, owning 2 properties (aOR 1.292, 95% CI 1.187‐1.406) and owning 3 or more properties (aOR 1.492, 95% CI 1.339‐1.664) were also associated with higher odds.

In the education access and quality domain, compared with junior high school or below, high school or vocational education (aOR 1.318, 95% CI 1.214‐1.432) and bachelor’s degree or above (aOR 1.487, 95% CI 1.377‐1.607) were associated with higher odds of DHI use. Higher health literacy (aOR 1.100 per 1-SD increase, 95% CI 1.067‐1.133) and eHL (aOR 1.202 per 1-SD increase, 95% CI 1.165‐1.240) were independently associated with DHI use.

In the health care access and quality domain, having health insurance was associated with higher odds of DHI use (aOR 1.219, 95% CI 1.110‐1.340). Reporting difficulty paying medical expenses was also associated with higher odds (aOR 1.314, 95% CI 1.221‐1.414). Relative to participants enrolled in family doctor services, those who were aware of but not enrolled in such services (aOR 0.646, 95% CI 0.600‐0.696) and those who did not know about such services (aOR 0.502, 95% CI 0.465‐0.542) had lower odds of DHI use.

In the neighborhood and built environment domain, western-region residence (aOR 0.918, 95% CI 0.862‐0.978) and rural residence (aOR 0.942, 95% CI 0.891‐0.996) were associated with only modestly lower odds of DHI use. In the social and community context domain, greater perceived social support was associated with higher odds of DHI use (aOR 1.068 per 1-SD increase, 95% CI 1.042‐1.095), whereas neighborhood relationship quality showed little evidence of an association with DHI use (aOR 0.978 per 1-SD increase, 95% CI 0.954‐1.002).

### Model Diagnostics and Adjusted Predicted Probabilities

Model diagnostics showed no evidence of severe multicollinearity among the covariates. The maximum adjusted GVIF was 1.905, and adjusted GVIF values for key socioeconomic and capability-related variables, including income, education, health literacy, and eHL, were all below 1.5 (Table S1 in Multimedia Appendix 1).

This study improved the interpretation of effect sizes on an absolute scale; adjusted predicted probabilities and absolute differences were estimated for selected determinants using marginal standardization (Figure 3; Table S2 in Multimedia Appendix 1). Meaningful absolute differences were observed for several socioeconomic, capability-related, and health system factors. The largest positive differences were observed for bachelor’s degree or above vs junior high school or below education (+8.96 percentage points), monthly income of 6001 CNY or more vs ≤3000 CNY or less (+7.16 percentage points), difficulty paying medical expenses vs no difficulty (+6.25 percentage points), and eHL at the 75th vs 25th percentile (+5.29 percentage points). In contrast, compared with participants enrolled in family doctor services, those who were aware but not enrolled and those unaware of such services had 10.20- and 15.91%-point lower adjusted probabilities, respectively. The adjusted urban-rural difference was small (−1.36 percentage points).

### Supplementary Stratified Analyses

Supplementary age- and region-stratified analyses are shown in Tables S3 and S4 in Multimedia Appendix 1. Overall, the direction of key associations was broadly consistent across subgroups for income, educational attainment, eHL, and family doctor service status. Positive associations between eHL and DHI use were observed across all age and regional strata. Higher income and higher educational attainment also generally showed positive associations across age and regional strata, although the magnitude of associations varied.

The positive association between difficulty paying medical expenses and DHI use was generally consistent across regions, although the estimated associations varied across age strata. Urban-rural differences were less consistent across subgroups. Lower odds of DHI use among rural participants were observed in the 18‐29-year-of-age stratum and the eastern-region stratum, whereas estimates in the remaining strata were closer to the null. Because multiple stratified comparisons were conducted without formal adjustment for multiplicity, these isolated subgroup findings should be interpreted cautiously and regarded as exploratory rather than definitive evidence of age- or region-specific differences. Overall, these exploratory analyses indicated greater consistency across strata for socioeconomic resources, health-related capabilities, and health system engagement than for urban-rural residence.

## Discussion

### Principal Findings

Using a large national survey with stratified and quota-based sampling, this study examined social and structural patterning in broader self-reported DHI use among the general adult population in China, during a period of rapid digital health expansion. Overall, DHI use was not randomly distributed across the population but was patterned by socioeconomic resources, health-related capabilities, and health system engagement. Higher income, higher educational attainment, higher health literacy, and higher eHL were consistently associated with greater DHI use. Health insurance coverage and enrollment in family doctor services were also positively associated with DHI use, whereas a lack of awareness of family doctor services was associated with substantially lower use. Adjusted predicted probabilities showed that these associations represented meaningful absolute differences, particularly for family doctor service status, educational attainment, income, and eHL.

A notable finding was that difficulty paying medical expenses was associated with higher DHI use. This pattern should be interpreted cautiously and may reflect need-driven or substitutional use among individuals experiencing greater health care pressure. The adjusted association between urban-rural residence and DHI use was small. Subgroup analyses further suggested that urban-rural patterns varied across age groups and regions. Together, these findings show that socioeconomic resources, health-related capabilities, and health system engagement were more consistently associated with broad DHI use than urban-rural residence alone.

### Comparison With Previous Work

Building on previous studies that focused on specific digital health formats and selected population groups before the AI expansion in China [13,18,19], the present study used the SDoH framework to examine a broader set of socioeconomic, capability-related, health care access, geographic, and social-contextual factors within a single analytic structure. This framing helps shift the interpretation of DHI use from an individual technology-adoption behavior to a pattern shaped by resources, skills, health system access, and place-based context. A further contribution is the use of recent postpandemic data collected during a period of increasing integration of AI-enabled technologies into digital health services. The outcome in this study was intentionally broad and should not be interpreted as subtype-specific adoption. Nevertheless, it captures an important population-level threshold: whether individuals have had any prior engagement with digital health technologies. In a health system where digital and AI-enabled tools are increasingly incorporated into service delivery, understanding who enter these digital health pathways remains a necessary step before examining sustained use, quality of use, or health outcomes [30-32]. By adding adjusted predicted probabilities and absolute differences, this study also provides a more interpretable estimate of the practical magnitude of inequalities in DHI use.

### Socioeconomic and Capability-Based Inequities in DHI Use

Consistent with the SDoH framework, economic stability and education-related capabilities emerged as important factors associated with DHI use. Higher income and educational attainment were positively associated with DHI use in this study, echoing evidence from both high-income countries [33-35] and middle-income countries [18] that digital health adoption tends to favor socially advantaged groups. These socioeconomic gradients likely reflect not only differential access to digital technologies but also unequal opportunities to develop the skills required for effective engagement with digital health services [36].

Beyond formal education, health literacy and eHL showed consistent positive associations with DHI use. Similar findings regarding the importance of eHL have been reported in previous studies, suggesting that differences in health-related capabilities may be relevant for understanding patterns of digital health use [11,37]. The consistency of the eHL association across age and regional subgroups suggests that capability-related barriers remain central in a rapidly digitalizing health system [38,39]. These findings align with the concept of a “second-level digital divide,” in which disparities arise from differences in skills and meaningful use rather than from access alone [40]. Therefore, accessing digital health technologies alone is insufficient to ensure equitable usage; individuals must also possess the cognitive and practical skills required to search for, interpret, and apply digital health information effectively [11].

### Health Care Access, Financial Pressure, and Health-System Engagement

Health care access indicators were associated with DHI use behavior in this study. Consistent with prior literature [11,12,33], having health insurance coverage was associated with DHI use, reflecting the role of financial protection in facilitating engagement with health care services. More prominently, enrollment in family doctor services showed one of the largest absolute differences in adjusted predicted probabilities. Prior research in China has shown that contracting with a family doctor is associated with enhancements in health status [41], highlighting the potential of primary care as an institutional interface. This finding should be interpreted in the context of the ongoing development of the family doctor system in China, where primary care-led digital health integration remains at an early and uneven stage [42,43].

The positive association between difficulty paying medical expenses and DHI use requires careful interpretation. This finding should not be taken to mean that financial hardship promotes equitable access to digital health; it may reflect need-driven use among individuals with greater perceived health care needs or financial pressure [44]. It may also reflect substitutional use, whereby people facing barriers to conventional care turn to digital tools because they are perceived as more convenient, lower-cost, or easier to access. Residual confounding by disease burden, health care usage frequency, or unmeasured health care needs may also contribute to this association. Because the study was cross-sectional and the DHI outcome was broad, we cannot determine whether financial difficulty preceded DHI use, whether DHI use was driven by specific lower-cost services, or whether this association varied by DHI subtype.

Together, these findings suggest that digital health equity depends not only on individual willingness or capability but also on how digital services are embedded within health care systems. Primary care, insurance coverage, and service awareness may be important leverage points for ensuring that digital health technologies reach groups who might otherwise be excluded.

### Geographic Disparities and the Limited Role of Community Context

Geographic patterns were less consistent than socioeconomic, capability-related, and health system factors. Although provincial variation in DHI use was observed, the adjusted associations for region and urban-rural residence were small, in contrast to the more pronounced geographic differences reported in some previous studies [12,33,45-47]. The adjusted urban-rural difference was particularly small in predicted probability analyses. Exploratory stratified analyses showed lower odds of DHI use among rural participants in the 18‐29-year-of-age stratum and the eastern-region stratum, whereas estimates in the remaining strata were closer to the null. Because multiple subgroup comparisons were conducted without formal adjustment for multiplicity, these isolated findings may reflect chance variation and should not be interpreted as definitive evidence of age- or region-specific differences.

Part of the urban-rural difference may be captured by socioeconomic resources, education, eHL, health care access, and regional context in the fully adjusted model. The binary ever-use outcome may be insensitive to differences in frequency, intensity, quality, purpose, or benefits of DHI use. Rural and urban residents may both report prior use, but their experiences with service quality, continuity, affordability, and effectiveness may differ substantially. The offline-to-online survey approach may also have underrepresented individuals who are most digitally excluded. As a result, urban-rural and regional disparities may have been underestimated.

An additional interesting finding is the differential association between social support and neighborhood relationship quality. Perceived social support was associated with modestly higher odds of DHI use, whereas neighborhood relationship quality showed little evidence of an association. This pattern may indicate that functional social resources are more relevant to digital health engagement than broader contextual or relational perceptions of the local community. From an SDoH perspective, social support may reflect access to informational, emotional, or instrumental resources that possibly facilitate engagement with health services [27,28], including digital platforms, whereas positive neighborhood relations alone may not translate into concrete support for digital health use. This finding highlights the importance of distinguishing between different dimensions of social context when examining inequities in digital health utilization.

### Implications for Digital Health Equity in China and Beyond

These findings carry important implications for digital health policy, implementation, and design in China and other rapidly digitalizing health systems. First, expanding digital health infrastructure alone is unlikely to ensure equitable use. Socioeconomic and capability-related gradients suggest that digital health strategies should incorporate literacy-sensitive and capability-sensitive design, including simplified interfaces, clear language, visual guidance, assisted onboarding, and support for users with limited digital experience. Second, digital health services should be integrated with trusted health system interfaces, particularly primary care and family doctor services. Such integration may help reduce informational barriers and provide human support for digital engagement.

Third, the positive association between difficulty paying medical expenses and DHI use suggests that digital health may be used by some individuals as a response to unmet or costly health care needs. Equity-oriented policies should therefore ensure that digital health does not become a lower-quality substitute for accessible in-person care among financially pressured groups. Digital pathways should be complemented by affordable, high-quality, and nondigital service options [44]. Fourth, place-based strategies remain important. Even though urban-rural residence showed only a small adjusted association in this study, regional variation and exploratory subgroup patterns suggest that digital health implementation should account for local infrastructure, primary care capacity, reimbursement arrangements, and population digital readiness.

Finally, as AI-enabled tools become part of digital health services, equity considerations should be incorporated into implementation and evaluation. The association between eHL and DHI use suggests that groups with lower digital and health-related capabilities may face barriers not only to accessing but also to understanding and evaluating digital or AI-generated health information. Human-centered design, transparent evaluation, and supported use through trusted health system interfaces may help prevent AI-enabled digital health services from reproducing existing inequities [31].

### Limitations

Several limitations should be noted. First, the cross-sectional design precludes causal inference regarding the observed associations. The observed associations should be interpreted as conditional associations rather than evidence that specific social determinants caused DHI use. Second, DHI use was measured as a binary self-reported outcome. The outcome captured broader DHI engagement and did not distinguish between DHI subtypes. It also did not capture frequency, duration, intensity, quality, or health impact of use. This may have obscured subtype-specific inequalities and limited the ability to identify which forms of DHI were most strongly associated with specific social determinants. Third, all measures were self-reported and therefore subject to reporting bias. Fourth, although the study was guided by the SDoH framework, some domains were necessarily operationalized using proxy indicators due to data constraints; these proxies may not fully capture more granular contextual features. Fifth, the analyses were not fully design-based weighted analyses. Individual-level selection probabilities and design weights were not available for all sampling stages, and the estimates should therefore be interpreted as adjusted associations within the final analytic sample. Sixth, the age- and region-stratified analyses were exploratory and involved multiple subgroup comparisons without formal adjustment for multiplicity. Isolated subgroup findings may therefore reflect chance variation and should not be interpreted as definitive evidence of effect modification by age or region. Finally, although the survey used stratified and quota-based sampling with broad geographic coverage, the analytic sample included a relatively high proportion of younger and urban participants; in addition, the offline-to-online data collection approach may have underrepresented individuals with limited digital access, low digital literacy, severe functional limitations, or lower willingness to complete electronic questionnaires [48], which may have attenuated observed disparities in DHI use.

### Conclusions

In this large national cross-sectional study, DHI use was patterned by socioeconomic resources, health-related capabilities, and health system engagement. Higher income, higher educational attainment, higher health literacy and eHL, health insurance coverage, and family doctor service enrollment were associated with greater DHI use, whereas the adjusted urban-rural difference was small. These findings suggest that equitable digital health transformation requires more than technological expansion. Policies and interventions should strengthen health and eHL, integrate digital health with accessible primary care, preserve nondigital support pathways, and apply equity-oriented design and governance principles, particularly as AI-enabled health tools become more widely implemented.

## Supplementary material

10.2196/94788 Multimedia Appendix 1 Supplementary sampling methods and analyses, including multicollinearity diagnostics, the primary model forest plot, predicted probabilities, and age- and region-stratified results.

## Abbreviations

aOR: adjusted odds ratio
DHI: digital health intervention
GI-AI4H: Global Initiative on AI for Health
GVIF: generalized variance inflation factors
PBICR: Psychology and Behavior Investigation of Chinese Residents
SDoH: Social Determinants of Health
STROBE: Strengthening the Reporting of Observational Studies in Epidemiology
WHO: World Health Organization
