# Supplementary material for: Social Determinants of Digital Health Intervention Use in China: National Cross-Sectional Study
Source: J Med Internet Res. 2026 Aug 21;28:e94788. doi: 10.2196/94788 (PMC13503648; doi:10.2196/94788)
Supplement: Supplementary file 1 [file jmir-v28-e94788-s001.docx]

# **Supplementary material**

Contents

[**Supplementary material**	1](#_Toc235627196)

[**1. Stratified and quota sampling method**	1](#_Toc235627197)

[**2. Multicollinearity diagnostics**	2](#_Toc235627198)

[**3. Figure S1 Factors associated with digital health intervention (DHI) use: adjusted odds ratios from multivariable logistic regression.**	3](#_Toc235627199)

[**4. Adjusted predicted probabilities**	4](#_Toc235627200)

[**5. Subgroup analyses**	5](#_Toc235627201)

## **1. Stratified and quota sampling method**

The data for this study were sourced from the 2024 Psychology and Behavior Investigation of Chinese Residents. A comprehensive three-stage sampling procedure was employed to enhance the national coverage and demographic balance of the sample [1].

Stage 1. Geographic Sampling: Four municipalities (Beijing, Tianjin, Shanghai, Chongqing), two special administrative regions (Hong Kong and Macao), and 150 cities were selected from 22 provinces and 5 autonomous regions. The number of cities chosen from each region was based on population size, with 2 to 12 cities randomly selected using a random number table. This step ensured broad geographic representation. Stage 2. Community Sampling: In this stage, 800 communities were selected proportionally from the 150 cities, with a 3:2 urban-to-rural ratio. The number of communities in each city was based on the population of the corresponding administrative region. Stage 3. Quota Sampling: Finally, quota sampling was used within each community to match gender and age distributions with China’s national population pyramid, maintaining a 1:1 gender ratio and reflecting the age structure [2]. This ensured demographic representation across the sample.

The survey was conducted from June 23 to September 29, 2024, covering 150 cities across 22 provinces, 5 autonomous regions, 4 municipalities, and 2 special administrative regions. Additionally, the study involved three rounds of pre-experiments, conducted between May 25 and May 28, May 30 and June 2, and June 4 and June 8, 2024. In each round, quota sampling was applied with the same demographic quotas as the formal survey. The pre-experiment samples consisted of 100 participants in the first and second rounds, and 200 participants in the third round. During this phase, feedback from respondents was collected, analyzed, and discussed by the research team, leading to revisions of the final questionnaire. Notably, data from the pre-surveys were not included in the final analysis. A detailed description of the PBICR 2024 study protocol has been reported elsewhere [1].

1.	Fan S, Li Y, Dong F, Chen W, Han Q, Duan T, Zhang J, Sun Q, Bao Y, Li Y, Zang S, Zhou X, Jiang F, Ming W kit, Sun X, Wu Y. Study protocol: A national cross-sectional study on psychology and behaviour investigation of Chinese residents in 2024, PBICR. medRxiv; 2025. p. 2025.01.14.25320520. doi: 10.1101/2025.01.14.25320520

2.	Wu Y, Fan S, Liu D, Sun X. Psychological and behavior investigation of Chinese residents: Concepts, practices, and prospects. Chinese General Practice Journal 2024 Sept 1;1(3):149–156. doi: 10.1016/j.cgpj.2024.07.006

## **2. Multicollinearity diagnostics**

**Table S1. Multicollinearity diagnostics for variables included in the multivariable logistic regression model**

| **Variable** | **Df** | **GVIF** | **Adjusted GVIF** |
| --- | --- | --- | --- |
| Age | 1 | 3.628 | 1.905 |
| Gender | 1 | 1.032 | 1.016 |
| Ethnicity | 1 | 1.258 | 1.122 |
| Religious belief | 1 | 1.089 | 1.044 |
| Marital status | 1 | 2.337 | 1.529 |
| Health status | 1 | 1.353 | 1.163 |
| Chronic disease | 1 | 1.294 | 1.138 |
| Monthly income | 2 | 1.246 | 1.057 |
| Debt status | 1 | 1.093 | 1.045 |
| Occupation | 3 | 3.580 | 1.237 |
| Property ownership | 4 | 1.243 | 1.028 |
| Education | 2 | 1.578 | 1.121 |
| Health literacy | 1 | 1.687 | 1.299 |
| eHealth literacy | 1 | 1.817 | 1.348 |
| Health insurance | 1 | 1.052 | 1.026 |
| Difficulty paying medical expenses | 1 | 1.118 | 1.057 |
| Family doctor service | 2 | 1.120 | 1.029 |
| Insurance location | 2 | 1.091 | 1.022 |
| Region | 2 | 1.240 | 1.055 |
| Residence | 1 | 1.129 | 1.063 |
| Social support | 1 | 1.218 | 1.104 |
| Neighborhood relationship | 1 | 1.247 | 1.117 |
| ***Notes:*** *Multicollinearity diagnostics were conducted for all variables included in the primary unweighted multivariable logistic regression model. For categorical variables with more than two levels, generalized variance inflation factors were reported. Adjusted GVIF was calculated as GVIF^(1/(2×Df)). All adjusted GVIF values were below 5, indicating no evidence of severe multicollinearity. Abbreviations: Df, degrees of freedom; GVIF, generalized variance inflation factor.* | | | |

## **3. Figure S1. Factors associated with digital health intervention (DHI) use: adjusted odds ratios from multivariable logistic regression.**

## **4. Adjusted predicted probabilities**

**Table S2. Adjusted predicted probabilities of any self-reported DHI use for selected social determinants**

| **Determinant** | **Level** | **Adjusted predicted probability, % (95% CI)** |
| --- | --- | --- |
| Monthly income, CNY | ≤3000 | 37.84 (36.81-38.88) |
| Monthly income, CNY | 3001-6000 | 42.27 (41.49-43.06) |
| Monthly income, CNY | ≥6001 | 45.00 (44.06-45.94) |
| Education | Junior high school or below | 35.17 (33.73-36.62) |
| Education | High school or vocational school | 41.34 (40.23-42.46) |
| Education | Bachelor’s degree or above | 44.13 (43.44-44.83) |
| Health literacy | P25 | 40.77 (40.13-41.41) |
| Health literacy | P75 | 42.37 (41.85-42.89) |
| eHealth literacy | P25 | 38.82 (38.08-39.56) |
| eHealth literacy | P75 | 44.11 (43.48-44.75) |
| Health insurance | No | 37.86 (35.86-39.86) |
| Health insurance | Yes | 42.29 (41.77-42.81) |
| Difficulty paying medical expenses | No | 41.31 (40.78-41.84) |
| Difficulty paying medical expenses | Yes | 47.56 (45.98-49.14) |
| Family doctor service | Yes | 53.30 (51.75-54.85) |
| Family doctor service | No, but aware of the service | 43.10 (42.37-43.83) |
| Family doctor service | Don’t know | 37.39 (36.59-38.20) |
| Residence | Urban | 42.32 (41.74-42.91) |
| Residence | Rural | 40.97 (39.89-42.05) |
| ***Notes***: *Adjusted predicted probabilities were estimated from the primary unweighted multivariable logistic regression model using marginal standardization. Estimates are presented as percentages. For health literacy, P25 and P75 correspond to raw values of 25.00 and 33.33, respectively. For eHealth literacy, P25 and P75 correspond to raw values of 15.00 and 21.00, respectively. Abbreviations: CI, confidence interval; CNY, Chinese yuan renminbi; DHI, digital health intervention. Exchange rate: CNY 1 ≈ US $0.148.* | | |

## **5. Subgroup analyses**

| **Table S3. Age-stratified associations between social determinants and digital health intervention use** | | | | | | |
| --- | --- | --- | --- | --- | --- | --- |
| **Domain** | **Characteristic** | **Classification** | **18-29 y aOR (95% CI)** | **30-44 y aOR (95% CI)** | **45-59 y aOR (95% CI)** | **≥60 y aOR (95% CI)** |
| **Sociodemographic covariates** | **Gender** | Female | 1.00 (Reference) | 1.00 (Reference) | 1.00 (Reference) | 1.00 (Reference) |
|  |  | Male | 1.057 (0.992–1.125) | 1.113 (1.006–1.231) | 1.135 (1.017–1.266) | 1.161 (0.991–1.360) |
|  | **Ethnicity** | Others | 1.00 (Reference) | 1.00 (Reference) | 1.00 (Reference) | 1.00 (Reference) |
|  |  | Han | 1.136 (1.027–1.258) | 1.018 (0.830–1.250) | 1.119 (0.857–1.471) | 0.923 (0.648–1.324) |
|  | **Religious belief** | No | 1.00 (Reference) | 1.00 (Reference) | 1.00 (Reference) | 1.00 (Reference) |
|  |  | Yes | 1.042 (0.922–1.177) | 0.871 (0.735–1.034) | 0.926 (0.766–1.121) | 1.363 (1.056–1.770) |
|  | **Marital status** | Married | 1.00 (Reference) | 1.00 (Reference) | 1.00 (Reference) | 1.00 (Reference) |
|  |  | Unmarried | 1.065 (0.923–1.230) | 1.402 (1.237–1.590) | 1.408 (1.188–1.668) | 1.162 (0.941–1.430) |
| **health-related covariates** | **Self-rated health** | Per 1-SD | 0.996 (0.962–1.031) | 0.942 (0.887–1.000) | 0.964 (0.903–1.030) | 0.976 (0.888–1.072) |
|  | **Chronic condition** | No | 1.00 (Reference) | 1.00 (Reference) | 1.00 (Reference) | 1.00 (Reference) |
|  |  | Yes | 1.253 (1.112–1.412) | 1.116 (0.971–1.281) | 1.087 (0.967–1.221) | 1.230 (1.031–1.469) |
| **Economic stability** | **Monthly income (CNY)** | ≤3,000 | 1.00 (Reference) | 1.00 (Reference) | 1.00 (Reference) | 1.00 (Reference) |
|  |  | 3,001-6,000 | 1.115 (1.032–1.204) | 1.315 (1.138–1.521) | 1.332 (1.154–1.539) | 1.459 (1.205–1.768) |
|  |  | ≥6,001 | 1.251 (1.147–1.364) | 1.461 (1.253–1.704) | 1.582 (1.345–1.862) | 1.377 (1.085–1.747) |
|  | **Debt status** | No | 1.00 (Reference) | 1.00 (Reference) | 1.00 (Reference) | 1.00 (Reference) |
|  |  | Yes | 1.003 (0.941–1.068) | 1.018 (0.918–1.129) | 0.996 (0.886–1.120) | 1.116 (0.900–1.381) |
|  | **Occupational status** | Employed | 1.00 (Reference) | 1.00 (Reference) | 1.00 (Reference) | 1.00 (Reference) |
|  |  | Student | 0.998 (0.920–1.084) | 1.456 (1.134–1.874) | 2.391 (1.619–3.572) | 1.082 (0.685–1.712) |
|  |  | Unemployed | 0.868 (0.694–1.084) | 0.959 (0.745–1.229) | 1.089 (0.881–1.341) | 0.938 (0.707–1.241) |
|  |  | Retired | 1.464 (0.765–2.845) | 1.237 (0.714–2.129) | 1.150 (0.966–1.368) | 1.043 (0.851–1.282) |
|  | **Number of properties** | None | 1.00 (Reference) | 1.00 (Reference) | 1.00 (Reference) | 1.00 (Reference) |
|  |  | 1 | 1.146 (1.042–1.261) | 1.039 (0.880–1.228) | 1.049 (0.833–1.327) | 1.174 (0.829–1.681) |
|  |  | 2 | 1.267 (1.135–1.414) | 1.375 (1.136–1.664) | 1.263 (0.986–1.623) | 1.941 (1.340–2.836) |
|  |  | ≥3 | 1.529 (1.330–1.758) | 1.659 (1.292–2.134) | 1.394 (1.024–1.899) | 1.592 (0.999–2.545) |
|  |  | Not sure | 1.034 (0.919–1.163) | 0.962 (0.724–1.274) | 1.472 (1.035–2.094) | 1.651 (1.062–2.577) |
| **Education access and quality** | **Education level** | Junior high school or below | 1.00 (Reference) | 1.00 (Reference) | 1.00 (Reference) | 1.00 (Reference) |
|  |  | High school or vocational school | 1.258 (1.007–1.576) | 1.022 (0.866–1.206) | 1.219 (1.050–1.415) | 1.543 (1.256–1.894) |
|  |  | Bachelor’s degree or above | 1.307 (1.056–1.623) | 1.375 (1.190–1.590) | 1.530 (1.326–1.765) | 1.641 (1.299–2.072) |
|  | **Health literacy index** | Per 1-SD | 1.104 (1.059–1.151) | 1.043 (0.977–1.113) | 1.150 (1.070–1.236) | 1.061 (0.954–1.179) |
|  | **eHealth literacy** | Per 1-SD | 1.118 (1.070–1.169) | 1.245 (1.159–1.338) | 1.258 (1.168–1.356) | 1.356 (1.225–1.502) |
| **Health care access and quality** | **Health insurance** | No | 1.00 (Reference) | 1.00 (Reference) | 1.00 (Reference) | 1.00 (Reference) |
|  |  | Yes | 1.380 (1.235–1.543) | 0.990 (0.763–1.287) | 0.889 (0.653–1.215) | 1.016 (0.666–1.564) |
|  | **Difficulty paying medical expenses** | No | 1.00 (Reference) | 1.00 (Reference) | 1.00 (Reference) | 1.00 (Reference) |
|  |  | Yes | 1.374 (1.241–1.522) | 1.116 (0.948–1.312) | 1.174 (0.983–1.399) | 1.656 (1.282–2.135) |
|  | **Family doctor service** | Yes | 1.00 (Reference) | 1.00 (Reference) | 1.00 (Reference) | 1.00 (Reference) |
|  |  | No, but aware of the service | 0.643 (0.570–0.724) | 0.744 (0.641–0.864) | 0.702 (0.600–0.821) | 0.560 (0.445–0.705) |
|  |  | Don’t know | 0.509 (0.451–0.574) | 0.601 (0.512–0.705) | 0.523 (0.443–0.618) | 0.432 (0.344–0.543) |
|  | **Location of basic medical insurance enrollment** | Local (current residence) | 1.00 (Reference) | 1.00 (Reference) | 1.00 (Reference) | 1.00 (Reference) |
|  |  | Place of household registration | 0.952 (0.890–1.018) | 0.988 (0.880–1.110) | 0.884 (0.783–0.998) | 0.818 (0.687–0.972) |
|  |  | Other locations | 0.965 (0.878–1.060) | 0.905 (0.716–1.141) | 1.184 (0.885–1.579) | 1.474 (0.950–2.294) |
| **Neighborhood and built environment** | **Region** | Eastern | 1.00 (Reference) | 1.00 (Reference) | 1.00 (Reference) | 1.00 (Reference) |
|  |  | Central | 1.012 (0.942–1.086) | 0.977 (0.870–1.097) | 1.143 (1.010–1.294) | 1.075 (0.894–1.294) |
|  |  | Western | 0.921 (0.846–1.003) | 0.858 (0.744–0.990) | 1.018 (0.869–1.191) | 1.012 (0.798–1.282) |
|  | **Residential type** | Urban | 1.00 (Reference) | 1.00 (Reference) | 1.00 (Reference) | 1.00 (Reference) |
|  |  | Rural | 0.863 (0.799–0.932) | 1.049 (0.921–1.194) | 1.102 (0.964–1.259) | 0.889 (0.742–1.065) |
| **Social and community context** | **Social support** | Per 1-SD | 1.082 (1.047–1.119) | 1.112 (1.052–1.176) | 1.054 (0.992–1.121) | 0.978 (0.892–1.072) |
|  | **Neighborhood relationship** | Per 1-SD | 0.999 (0.966–1.032) | 0.970 (0.918–1.025) | 0.952 (0.895–1.013) | 1.001 (0.913–1.097) |

***Note****: Abbreviations: aOR, adjusted odds ratio; CI, confidence interval; SD, standard deviation. Models were fitted separately within each age subgroup using the same covariates as the primary model, except age. Continuous variables are interpreted per 1-SD increase. Exchange rate: CNY 1 ≈ US $0.148.*

| **Table S4. Region-stratified associations between social determinants and digital health intervention use** | | | | | |
| --- | --- | --- | --- | --- | --- |
| **Domain** | **Characteristic** | **Classification** | **Eastern China, aOR (95% CI)** | **Central China, aOR (95% CI)** | **Western China, aOR (95% CI)** |
| **Sociodemographic covariates** | **Age** | Per 1-SD | 0.954 (0.892–1.019) | 0.892 (0.829–0.960) | 0.860 (0.776–0.954) |
|  | **Gender** | Female | 1.00 (Reference) | 1.00 (Reference) | 1.00 (Reference) |
|  |  | Male | 1.066 (0.991–1.146) | 1.108 (1.030–1.192) | 1.096 (0.995–1.208) |
|  | **Ethnicity** | Others | 1.00 (Reference) | 1.00 (Reference) | 1.00 (Reference) |
|  |  | Han | 1.032 (0.878–1.215) | 1.112 (0.907–1.367) | 1.152 (1.025–1.296) |
|  | **Religious belief** | No | 1.00 (Reference) | 1.00 (Reference) | 1.00 (Reference) |
|  |  | Yes | 0.857 (0.754–0.975) | 1.210 (1.027–1.428) | 1.038 (0.897–1.203) |
|  | **Marital status** | Married | 1.00 (Reference) | 1.00 (Reference) | 1.00 (Reference) |
|  |  | Unmarried | 1.397 (1.258–1.550) | 1.236 (1.097–1.392) | 1.245 (1.069–1.449) |
| **Health-related covariates** | **Self-rated health** | Per 1-SD | 0.955 (0.916–0.996) | 1.013 (0.971–1.057) | 0.951 (0.903–1.003) |
|  | **Chronic condition** | No | 1.00 (Reference) | 1.00 (Reference) | 1.00 (Reference) |
|  |  | Yes | 1.104 (0.996–1.224) | 1.189 (1.070–1.322) | 1.174 (1.019–1.352) |
| **Economic stability** | **Monthly income (CNY)** | ≤3000 | 1.00 (Reference) | 1.00 (Reference) | 1.00 (Reference) |
|  |  | 3001-6000 | 1.191 (1.073–1.322) | 1.226 (1.122–1.341) | 1.228 (1.096–1.377) |
|  |  | ≥6001 | 1.409 (1.264–1.571) | 1.275 (1.149–1.414) | 1.395 (1.219–1.595) |
|  | **Debt status** | No | 1.00 (Reference) | 1.00 (Reference) | 1.00 (Reference) |
|  |  | Yes | 1.167 (1.082–1.259) | 0.924 (0.855–0.998) | 0.960 (0.869–1.060) |
|  | **Occupational status** | Employed | 1.00 (Reference) | 1.00 (Reference) | 1.00 (Reference) |
|  |  | Student | 1.034 (0.929–1.151) | 0.943 (0.840–1.058) | 1.090 (0.941–1.262) |
|  |  | Unemployed | 1.033 (0.842–1.264) | 0.998 (0.828–1.200) | 0.849 (0.680–1.055) |
|  |  | Retired | 1.055 (0.887–1.253) | 1.098 (0.929–1.296) | 1.438 (1.131–1.827) |
|  | **Number of properties** | None | 1.00 (Reference) | 1.00 (Reference) | 1.00 (Reference) |
|  |  | 1 | 1.074 (0.952–1.212) | 1.098 (0.967–1.246) | 1.054 (0.907–1.226) |
|  |  | 2 | 1.293 (1.133–1.477) | 1.347 (1.170–1.553) | 1.241 (1.037–1.487) |
|  |  | ≥3 | 1.440 (1.221–1.698) | 1.638 (1.363–1.971) | 1.397 (1.097–1.780) |
|  |  | Not sure | 1.060 (0.903–1.244) | 1.139 (0.968–1.340) | 0.950 (0.773–1.167) |
| **Education access and quality** | **Education level** | Junior high school or below | 1.00 (Reference) | 1.00 (Reference) | 1.00 (Reference) |
|  |  | High school or vocational school | 1.255 (1.095–1.438) | 1.375 (1.211–1.561) | 1.264 (1.052–1.519) |
|  |  | Bachelor’s degree or above | 1.412 (1.244–1.604) | 1.557 (1.377–1.760) | 1.448 (1.224–1.716) |
|  | **Health literacy index** | Per 1-SD | 1.133 (1.081–1.189) | 1.070 (1.020–1.124) | 1.101 (1.033–1.173) |
|  | **eHealth literacy** | Per 1-SD | 1.190 (1.131–1.253) | 1.253 (1.190–1.319) | 1.145 (1.071–1.224) |
| **Health care access and quality** | **Health insurance** | No | 1.00 (Reference) | 1.00 (Reference) | 1.00 (Reference) |
|  |  | Yes | 1.255 (1.084–1.454) | 1.245 (1.060–1.464) | 1.185 (0.975–1.442) |
|  | **Difficulty paying medical expenses** | No | 1.00 (Reference) | 1.00 (Reference) | 1.00 (Reference) |
|  |  | Yes | 1.343 (1.187–1.518) | 1.268 (1.122–1.433) | 1.357 (1.180–1.561) |
|  | **Family doctor service** | Yes | 1.00 (Reference) | 1.00 (Reference) | 1.00 (Reference) |
|  |  | No, but aware of the service | 0.651 (0.585–0.725) | 0.708 (0.616–0.814) | 0.606 (0.517–0.710) |
|  |  | Don’t know | 0.510 (0.456–0.571) | 0.561 (0.487–0.647) | 0.454 (0.386–0.533) |
|  | **Location of basic medical insurance enrollment** | Local | 1.00 (Reference) | 1.00 (Reference) | 1.00 (Reference) |
|  |  | Place of household registration | 0.929 (0.854–1.011) | 0.921 (0.851–0.996) | 0.956 (0.864–1.057) |
|  |  | Other locations | 1.047 (0.922–1.190) | 0.962 (0.845–1.094) | 0.890 (0.736–1.074) |
| **Neighborhood and built environment** | **Residential type** | Urban | 1.00 (Reference) | 1.00 (Reference) | 1.00 (Reference) |
|  |  | Rural | 0.875 (0.796–0.962) | 0.946 (0.864–1.036) | 1.011 (0.907–1.127) |
| **Social and community context** | **Social support** | Per 1-SD | 1.038 (0.998–1.079) | 1.071 (1.029–1.116) | 1.123 (1.067–1.183) |
|  | **Neighborhood relationship** | Per 1-SD | 0.951 (0.913–0.989) | 1.021 (0.981–1.063) | 0.962 (0.914–1.013) |

***Note****: Abbreviations: aOR, adjusted odds ratio; CI, confidence interval; SD, standard deviation. Models were fitted separately within each region using the same covariates as the primary model, except region. Continuous variables are interpreted per 1-SD increase. Exchange rate: CNY 1 ≈ US $0.148.*
